# Exploring stachydrine: from natural occurrence to biological activities and metabolic pathways

**DOI:** 10.3389/fpls.2024.1442879

**Published:** 2024-08-06

**Authors:** Zekun He, Peng Li, Pan Liu, Ping Xu

**Affiliations:** ^1^ Shanghai Key Laboratory of Plant Functional Genomics and Resources, Shanghai Chenshan Botanical Garden, Shanghai Chenshan Plant Science Research Center, Chinese Academy of Sciences, Shanghai, China; ^2^ State Key Laboratory of Plant Molecular Genetics, Chinese Academy of Sciences (CAS) Center for Excellence in Molecular Plant Sciences, Chinese Academy of Sciences, Shanghai, China; ^3^ University of Chinese Academy of Sciences, Beijing, China

**Keywords:** stachydrine, *Leonurus japonicus*, pharmacological effects, cardioprotective, osmoprotective, biosynthesis pathway

## Abstract

Stachydrine, also known as proline betaine, is a prominent constituent of traditional Chinese herb *Leonurus japonicus*, renowned for its significant pharmacological effects. Widely distributed in plants like *Leonurus* and *Citrus aurantium*, as well as various bacteria, stachydrine serves pivotal physiological functions across animal, plant, and bacterial kingdoms. This review aims to summarizes diverse roles and mechanisms of stachydrine in addressing cardiovascular and cerebrovascular diseases, neuroprotection, anticancer activity, uterine regulation, anti-inflammatory response, obesity management, and respiratory ailments. Notably, stachydrine exhibits cardioprotective effects via multiple pathways encompassing anti-inflammatory, antioxidant, anti-apoptotic, and modulation of calcium handling functions. Furthermore, its anti-cancer properties inhibit proliferation and migration of numerous cancer cell types. With a bi-directional regulatory effect on uterine function, stachydrine holds promise for obstetrics and gynecology-related disorders. In plants, stachydrine serves as a secondary metabolite, contributing to osmotic pressure regulation, nitrogen fixation, pest resistance, and stress response. Similarly, in bacteria, it plays a crucial osmoprotective role, facilitating adaptation to high osmotic pressure environments. This review also addresses ongoing research on the anabolic metabolism of stachydrine. While the biosynthetic pathway remains incompletely understood, the metabolic pathway is well-established. A deeper understanding of stachydrine biosynthesis holds significance for elucidating its mechanism of action, advancing the study of plant secondary metabolism, enhancing drug quality control, and fostering new drug development endeavors.

## 1 Introduction

**Figure 1:**
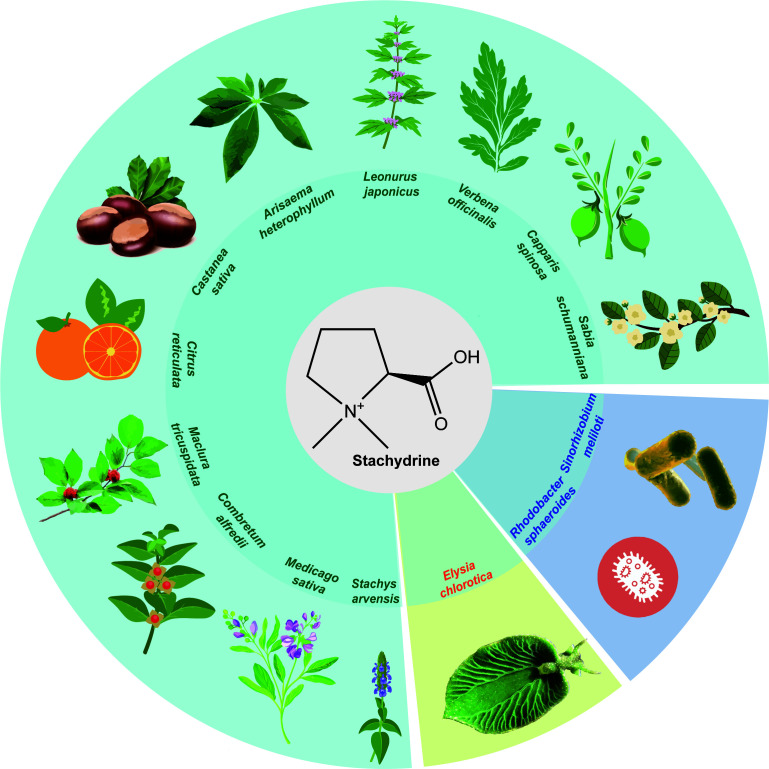
The natural occurrence of stachydrine. Stachydrine is found in the following plants: *Leonurus*, *Capparis spinosa*, *Castanea sativa*, *Citrus reticulata*, *Medicago sativa*, *Sabia schumanniana*, *Maclura tricuspidata*, *Verbena officinalis*, *Arisaema heterophyllum*, *Combretum alfredii*, and *Stachys arvensis*. In addition to these plants, stachydrine may also be found in other plants in the same genus as these plants. It is also found in the Elysia chlorotica and Sinorhizobium meliloti and Rhodobacter sphaeroides.

Stachydrine, also recognized as proline betaine and *N*, *N*-dimethyl-L-proline, represents an alkaloid characterized by the molecular structure of (2S)-1,1-dimethylpyrrolidine-2-carboxylic acid (shown in **Figure 1**). First isolated by Steenbock in 1918, stachydrine is noted for its biological activities and pharmaceutical potential (Connor et al., 1973). It is a key component of the traditional Chinese medicinal herb *Leonurus*, commonly known as motherwort, which is renowned for its pharmacological effectiveness (Kuchta et al., 2013).

*Leonurus*, belonging to the Labiatae family, is rich in alkaloids, among which stachydrine constitutes a significant proportion, ranging from 0.59% to 1.72% (Dai et al., 2016a). Revered in ancient medical texts such as “Shennong’s Classic of a Hundred Herbs,” *Leonurus*, classified as ‘superior’ and non-toxic, is extensively used across China, Korea, and Japan, primarily for managing gynecological disorders. Furthermore, it has been employed for centuries in European countries to address neurological and functional heart conditions (Li et al., 2024). According to the Chinese Pharmacopoeia, *Leonurus* manifests various therapeutic effects, including blood circulation activation, menstruation regulation, diuretic and anti-inflammatory properties, and heat-clearing detoxification (The Pharmacopoeia Committee, 2020). Its widespread use is evidenced by the annual sales of *Leonurus*-related products in China, which amount to approximately one billion RMB. Chemical analysis has identified over 140 components from *Leonurus*, predominantly alkaloids, flavonoids, and terpenoids, supplemented by substantial potassium and vitamin content (Shang et al., 2014). Alkaloids, such as leonurine, stachydrine, betaine, and trigonelline, have been recognized as the principal bioactive compounds within *Leonurus* (Zhang et al., 2018; Li et al., 2022a).

*Leonurus* is not the only plant that produces stachydrine; it is also found in *Capparis spinosa* (Maresca et al., 2016), *Castanea sativa* (Servillo et al., 2016), *Citrus reticulata (* Heinzmann et al., 2010), *Medicago sativa* (Connor et al., 1973), *Sabia schumanniana* (Gong et al., 2012), *Maclura tricuspidata* (State Administration of Medicine Chinese herbal medicine information center station, 1986), *Verbena officinalis* (Cheng et al., 2010), *Arisaema heterophyllum* (Yu and Yu, 2007), *Combretum alfredii* (Wu et al., 2007), and *Stachys arvensis* (Cheng et al., 2020). Additionally, bacteria such as *Sinorhizobium meliloti* (Phillips et al., 1998) in alfalfa and *Rhodobacter sphaeroides* (Kumar et al., 2014) also produce stachydrine (**Figure 1**). Among these, *Medicago sativa* and *Leonurus* are the most extensively studied, containing approximately 0.1% and 1% dry weight (DW) of stachydrine, respectively (Connor et al., 1973; Kuchta et al., 2014). *Citrus reticulata* contains about 0.3% (DW), with lower levels reported in other plants (Sun et al., 2023).

In addition to being synthesized in plants, stachydrine may also be produced in animals. One study noted that a mollusk, the *Elysia chlorotica*, produces its own stachydrine, which regulates the size of its cells through stachydrine (Pierce et al., 1984) (**Figure 1**). It is also found in the human gut and kidneys and plays an important role in regulating osmotic pressure, which is likely derived from human food intake (Chambers and Kunin, 1987a).

## 2 Therapeutic roles of stachydrine in humans

### 2.1 Cardiovascular and neurological effects

Cardiovascular and cerebrovascular diseases, collectively termed cardiovascular diseases, are the leading causes of death globally according to the World Health Organization. Cardiovascular diseases encompass various pathophysiological states such as atherosclerosis, acute myocardial infarction, chronic heart failure, and vasospasm, while cerebrovascular diseases include conditions like ischemic stroke, craniocerebral trauma, and neurodegenerative disorders such as Alzheimer’s disease, Parkinson’s disease, and depression. The crucial roles of the heart and brain in overall health make their impairments significantly detrimental.

Current pharmacological treatments predominantly rely on chemically synthesized small molecule drugs, including nitrates, statins, beta-blockers, clopidogrel, aspirin, and ACE inhibitors/angiotensin receptor blockers (ARBs). Although these treatments are effective, they often come with considerable side effects and costs. It is crucial to explore safer and more efficient therapeutic alternatives and comprehensive treatment protocols to improve therapeutic outcomes and patient prognosis.

Recent research shows that stachydrine, a plant-derived natural product, is highly effective in treating cardiovascular and cerebrovascular diseases.

#### 2.1.1 Cardiac cell regulation

It mitigates the enlargement of cardiac cells induced by various stimuli, such as norepinephrine and angiotensin II. Specifically, stachydrine impedes the calcium-modulated phosphatase/NFAT signaling cascade, thereby attenuating the aberrant cardiac hypertrophy elicited by adrenergic receptor activation. By disrupting the influence of these factors on cardiac cell signaling pathways, stachydrine effectively curtails pathological myocardial growth (Guo et al., 2012; Cao et al., 2017; Zheng et al., 2020). Furthermore, stachydrine confers cytoprotective effects by thwarting cardiomyocyte apoptosis triggered by hypoxia and regulating iron metabolism to mitigate iron-induced cell death (Liu et al., 2009; Liang et al., 2023). This protective mechanism is instrumental in combating conditions like myocardial ischemia and heart failure.

Stachydrine exerts regulatory control over calcium homeostasis within cardiac cells, thereby augmenting the efficiency of calcium transients while mitigating calcium leakage from cardiomyocytes. This regulatory action is essential for ensuring proper cardiac function during both the contraction and relaxation phases of the cardiac cycle (Li et al., 2022b).

Stachydrine augments the activity of key antioxidant enzymes, notably superoxide dismutase (SOD) and glutathione peroxidase (GSH-Px), while concurrently attenuating oxidative stress, lipid peroxidation, and the accumulation of reactive oxygen species (ROS). Through these mechanisms, stachydrine effectively counters the cellular damage induced by ischemia, reperfusion injury, or other oxidative insults. Consequently, stachydrine serves as a protective agent safeguarding the integrity of both the cardiovascular and cerebrovascular systems (Xie et al., 2018; Li et al., 2021; Lu et al., 2023).

#### 2.1.2 Inhibition and amelioration of myocardial fibrosis

Stachydrine effectively inhibits the production and activity of key mediators involved in myocardial fibrosis, notably Angiotensin II (AngII) and Transforming Growth Factor β1 (TGFβ1). It achieves this by downregulating the expression of angiotensinogen (AGT) and angiotensin-converting enzyme (ACE) within cardiac tissues. These actions are crucial for halting the progression of cardiac fibrosis. TGFβ1, in particular, plays a central role in the pathogenesis of cardiac fibrosis. Stachydrine prevents the transformation of cardiac fibroblasts into myofibroblasts, a critical step in fibrosis, by interfering with this pathway. Importantly, stachydrine also modulates the TGF-β/Smad signaling axis, which is instrumental in activating fibroblasts and synthesizing collagen in the heart. By blocking the phosphorylation of Smad proteins, stachydrine reduces the expression of pro-fibrotic genes, thus diminishing the fibrotic response. Additionally, stachydrine directly inhibits cardiac fibroblast proliferation and activation triggered by AngII. This leads to a decrease in collagen production and other extracellular matrix components, thereby significantly alleviating myocardial fibrosis (Chen et al., 2017; Liu et al., 2019).

#### 2.1.3 Inhibit inflammatory response

Stachydrine displays potent anti-inflammatory properties by decreasing the release of key inflammatory mediators, including interleukin-1β (IL-1β) and tumor necrosis factor-α (TNF-α). It also counteracts the NF-κB (nuclear factor κB) signaling pathway, a critical transcription factor that orchestrates intracellular inflammation. Stachydrine effectively blocks the phosphorylation and nuclear translocation of the NF-κB p65 subunit, thus reducing the production of inflammatory molecules. Additionally, it modulates the JAK/STAT (Janus kinase/signal transducer and activator of transcription) signaling pathway, which regulates cellular responses to cytokines and growth factors. Importantly, it inhibits the production of phosphorylated STAT3 (p-STAT3) and JAK2 (p-JAK2), reducing the transcriptional activity of inflammatory genes. This regulation of inflammation is crucial in managing cardiovascular diseases, as inflammation plays a central role in the development of atherosclerosis and other related complications. The comprehensive anti-inflammatory effects of stachydrine make it a promising candidate for treating inflammation-associated cardiovascular disorders (Zhao et al., 2017; Wu et al., 2020; Yu et al., 2023).

#### 2.1.4 Improvement of homocysteine-induced endothelial dysfunction

Hyperhomocysteinemia (HHcy) is recognized as an independent risk factor for cardiovascular disease. Stachydrine has shown effectiveness in alleviating the detrimental effects of homocysteine (Hcy) on endothelial vasodilation across various arterial segments, such as the thoracic aorta, mesenteric arteries, and renal arteries in rat models. It enhances the production of tetrahydrobiopterin (BH4), a crucial cofactor for endothelial nitric oxide synthase (eNOS), by upregulating the expression of GTP cyclohydrolase 1 (GTPCH1) and dihydrofolate reductase (DHFR) within endothelial cells. This increase in eNOS activity highlights the essential role of BH4 in maintaining endothelial function. These findings emphasize stachydrine’s potential as a therapeutic agent for mitigating endothelial dysfunction caused by Hcy, providing vasoprotective benefits. Furthermore, the study reveals a novel molecular mechanism by which stachydrine exerts its vasoprotective effects, offering insights into its promising applications for managing cardiovascular diseases (Xie et al., 2018).

#### 2.1.5 Promotes nitric oxide production and modulates n-glycosylation

Stachydrine boosts the production of nitric oxide (NO) by activating the AMPK and Akt signaling pathways, which in turn enhances the phosphorylation and activity of endothelial nitric oxide synthase (eNOS). Nitric oxide is a critical vasodilator that aids in the relaxation of blood vessels, thereby supporting healthy vascular function and preventing endothelial dysfunction (Xie et al., 2018; Xie et al., 2019).

Stachydrine modulates the *N*-glycosylation of the β1-adrenergic receptor (β1AR) by inhibiting α-1,6-fucosylation. This action preserves the normal functionality of β1AR and supports the excitation-contraction coupling process in the heart, crucial for maintaining cardiac rhythm and function (Hu et al., 2021).

#### 2.1.6 Inhibits of platelet activation and thrombosis

Stachydrine effectively reduces the risk of thrombosis by mitigating platelet activation, aggregation, and secretion. Additionally, it attenuates the interactions between platelets and neutrophils, decreasing the likelihood of inflammatory and thrombotic complications. These mechanisms enable stachydrine to improve cardiac function, reduce cardiac load, counteract cardiac remodeling, and, to a certain extent, reverse cardiac dysfunction. Consequently, stachydrine emerges as a promising cardioprotective agent with significant potential in the treatment of cardiovascular diseases (Mansurov, 1972; Sun et al., 2022).

#### 2.1.7 Neuroprotective actions

Stachydrine has demonstrated significant efficacy in reducing cell death, neurological impairment, and brain tissue damage in mouse models of traumatic brain injury (TBI). It notably decreases apoptosis, infarct volume, and brain water content in various experimental TBI models (Yu et al., 2018). Additionally, stachydrine promotes cellular growth, inhibits cell death, and alleviates inflammation, thus protecting neurons and ameliorating cognitive deficits in rat models of TBI (Yu et al., 2018). It also modulates key biomarkers of oxidative and inflammatory responses, including superoxide dismutase (SOD), malondialdehyde (MDA), interleukin-1β (IL-1β), and tumor necrosis factor-α (TNF-α). Furthermore, stachydrine reduces cerebral infarction size in rats subjected to middle cerebral artery occlusion and shields neurons from TBI-induced damage. These protective actions are mediated through the suppression of the PI3K/m-TOR/Akt and TLR4/NF-κB pathways, crucial regulators of cell survival, proliferation, and apoptosis. Through these mechanisms, stachydrine significantly enhances neurological function (Li et al., 2020).

### 2.2 Anticancer properties

Extensive research has consistently highlighted stachydrine’s potent antagonistic effects on various types of cancer, including astrocytoma (Liu et al., 2018), prostate cancer (Rathee et al., 2012), breast cancer (Bao et al., 2022), colon cancer (Zhao, 2018), gastric cancer (Ma et al., 2017), esophageal squamous cell carcinoma (Isozaki et al., 2014), chronic myeloid leukemia (CML) (Gu et al., 2022), hepatocellular carcinoma (HCC) (Chen and Yan, 2021), and others. Stachydrine’s anticancer activities primarily involve the inhibition of cell proliferation, induction of apoptosis, and blocking of cell migration and invasion. These effects are achieved through the modulation of multiple molecular pathways, providing a robust mechanistic foundation for its therapeutic potential against a wide array of cancers.

Stachydrine effectively inhibits several receptor tyrosine kinases, including BCR-ABL, which is crucial in the treatment of chronic myeloid leukemia (CML). By blocking these kinases, stachydrine disrupts proliferation and survival signaling in cancer cells, thereby curbing their growth and viability (Gu et al., 2022).

Stachydrine impacts multiple signaling pathways that are pivotal in cancer progression, such as PI3K/Akt, ERK/MAPK, and NF-κB. These pathways are integral to cell proliferation, survival, migration, and inflammatory responses. Stachydrine’s anticancer effects are mediated through the inhibition of key molecules within these pathways, including Akt, ERK, and IκBα, leading to reduced cancer cell activity (Liu et al., 2018).

Stachydrine promotes cancer cell apoptosis by activating pathways involved in programmed cell death, particularly the mitochondrial pathway. It lowers the mitochondrial membrane potential and activates key apoptotic proteins such as Bax and caspase-3, while simultaneously reducing the expression of the anti-apoptotic protein Bcl-2. This dual action both triggers apoptosis and inhibits survival signals within cancer cells (Bao et al., 2022; Zeng et al., 2023).

Stachydrine impedes the migration and invasive capabilities of tumor cells by inhibiting signaling pathways such as CXCR4/ERK and CXCR4/Akt. This inhibition is crucial for preventing the metastasis and spread of cancer, effectively blocking critical pathways involved in tumor cell dissemination (Liu et al., 2018).

Stachydrine modulates cell cycle progression, causing cancer cells to arrest in the G0/G1 phase, which hampers their proliferation. It has shown particular efficacy against corticoblastic astrocytoma (PA), inducing apoptosis and arresting cell cycle progression in human PA cells. These effects are mediated through the inhibition of CXCR4/Akt and CXCR4/ERK pathways and their downstream effectors, including CXCR4/Akt/MMP-9/2 and CXCR4/ERK/MMP-9/2, leading to decreased cell viability and reduced colony formation in PA cells (Liu et al., 2018; Zhai et al., 2024).

Stachydrine protects against oxidative stress by enhancing the activity of antioxidant enzymes such as superoxide dismutase (SOD). It also lowers serum lactate dehydrogenase levels, indicative of reduced oxidative damage. In studies on mice with gastric cancer induced by 1-methyl-3-nitro-1-nitrosoguanidine, stachydrine inhibited histone deacetylase (HDAC) activity in gastrointestinal tissues, significantly decreasing oxidative stress markers and cytokine levels. These findings suggest that stachydrine can mitigate oxidative damage in gastric cancer by inhibiting HDAC activity (Ma et al., 2017).

Despite its potent anticancer properties, stachydrine faces challenges in clinical implementation, particularly concerning bioavailability and pharmacokinetic properties. Researchers are actively working to enhance its therapeutic efficacy and safety profile through the synthesis of derivatives and the optimization of delivery techniques. These efforts aim to maximize stachydrine’s effectiveness in cancer treatment by improving its stability, absorption, and targeted delivery.

### 2.3 Effects of stachydrine on uterus

*Leonurus* has been traditionally used in the treatment of various obstetric and gynecological conditions. To evaluate its safety and efficacy, a study was conducted using a *Leonurus* injection, which contains 1 mL of solution with 18–22 mg of stachydrine hydrochloride, aimed at preventing post-abortion bleeding (Xia et al., 2020). The results confirmed its effectiveness in reducing post-abortion hemorrhage and promoting uterine retraction, thereby decreasing bleeding post-abortion. The injection also demonstrated a favorable safety profile, with no adverse reactions such as nausea, vomiting, diarrhea, or local reactions at the injection site, and no detrimental effects on liver and kidney functions were observed.

Stachydrine, a key compound found in *Leonurus*, exhibits a dual regulatory effect on uterine activity. In healthy guinea pigs, it enhances uterine contractions by increasing contraction intensity and decreasing contraction frequency (Dai et al., 2016b). Furthermore, it counteracts uterine contractions induced by oxytocin (Zheng and Wang, 2017). Stachydrine also inhibits abnormal proliferation of uterine smooth muscle cells (MSMC) induced by LPS stimulation, through the modulation of calcium-regulating proteins (Zeng, 2007). Its significant role in managing postpartum hemorrhage and aiding uterine recovery is highlighted by its ability to enhance uterine contraction and promote angiogenesis (He et al., 2018).

Despite the biological properties similar to those of *Leonurus*, research and application of stachydrine in obstetrics and gynecology remain relatively limited. Therefore, comprehensive future studies are essential to fully explore its potential applications and elucidate the underlying mechanisms in obstetric and gynecological contexts.

### 2.4 Anti-inflammatory effects of stachydrine

Research has identified stachydrine as an effective anti-arthritic and analgesic component in Capparis spinosa, demonstrating suitability for treating arthritis induced by Complete Freund’s Adjuvant (CFA) in rats (Feng et al., 2011). These findings support the anti-inflammatory properties of stachydrine. To further explore these properties, researchers have developed several inflammation models, especially those induced by Lipopolysaccharide (LPS). LPS, a major component of the cell wall of Gram-negative bacteria, triggers endothelial cells to overexpress inflammatory cytokines, initiating inflammation cascades that can lead to sepsis and multi-organ dysfunction (Dauphinee and Karsan, 2006).

Studies have shown that high doses of stachydrine can counteract endotoxin effects and inflammation, as evidenced by reduced hepatic and intestinal damage indices in mice with LPS-induced inflammation, without altering serum levels of LPS, TNF-α, and IL-1β. Additionally, stachydrine has been effective in inhibiting LPS-induced inflammatory bone loss by suppressing osteoclastogenesis both *in vitro* and *in vivo*. This effect is mediated by the inhibition of the NF-κB and AKT signaling pathways, activated by the receptor activator of NF-κB ligand (RANKL), highlighting its potential in treating conditions like osteoporosis (Meng et al., 2019).

Further, the anti-inflammatory activity of stachydrine was observed in various experimental models, including ear swelling in mice induced by xylene, granuloma formation from cotton ball implantation, and pleurisy in rats induced by carrageenan. These effects were linked to improvements in cell membrane permeability, suppression of inflammatory cytokine levels, and reduction in lipid peroxidation (Fang and Can, 2012).

**Figure 2:**
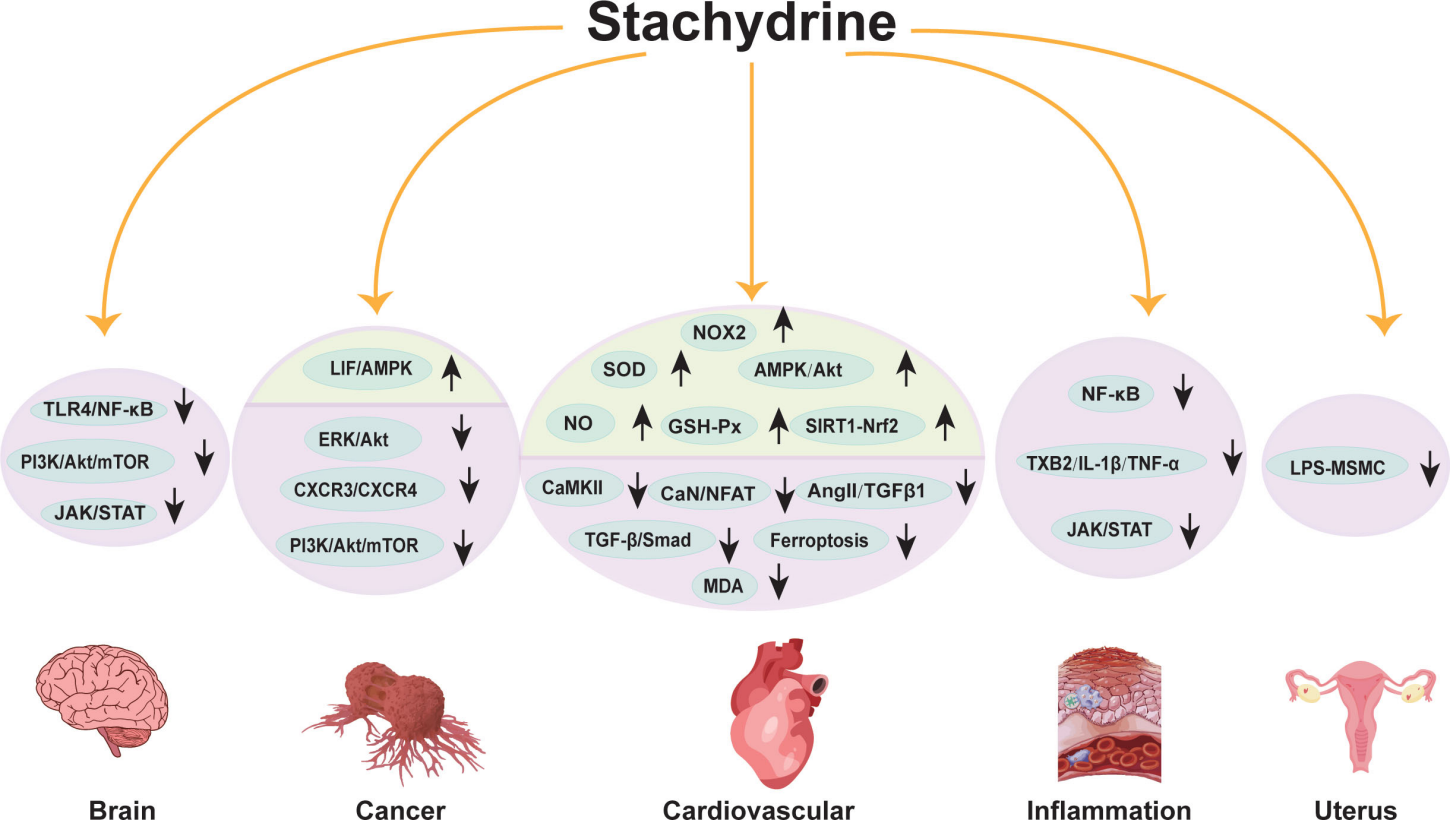
Pharmacological mechanisms of stachydrine. This figure outlines the pharmacological mechanisms of stachydrine, primarily affecting the cardiovascular system, cancer, inflammation, uterus, and nervous system. The direction of the arrows indicates the upregulation or downregulation of substances or signaling pathways.

**Figure 2** illustrates the above pharmacological mechanisms of stachydrine.

### 2.5 Other effects of stachydrine in humans

Stachydrine has shown promising therapeutic effects against obesity and insulin resistance. Korean researchers have identified properties in a commonly consumed rice wine that contribute to weight reduction and attributed these effects to stachydrine. It promotes lipid breakdown and prevents lipid accumulation in 3T3-L1 adipocytes, reduces weight gain, and improves glucose tolerance and insulin sensitivity in mouse models. Notably, stachydrine significantly decreases adipsin mRNA levels in both liver and adipose tissue, while increasing adipsin levels in the bloodstream of mice compared to those on a high-fat diet alone. It also restores balance in endoplasmic reticulum function and modulates adipsin expression, highlighting its potential as a therapeutic agent against obesity and insulin resistance (Lee et al., 2022).

Stachydrine also exhibits osmoprotective effects on human kidneys and the surrounding microbiota. Although the precise mechanism of stachydrine formation in the human body remains unclear, its extraction from human urine and subsequent studies indicates its role in providing osmoprotection. This protective effect is crucial for renal health and maintaining microbial balance (Chambers and Kunin, 1987a).

Additionally, stachydrine offers notable respiratory benefits, particularly in reducing cough frequency. This has been demonstrated in a guinea pig cough model induced by citric acid, where stachydrine targets sensory nerve endings in the respiratory tract, reducing the sensitivity of the cough reflex and exerting an antitussive effect. Furthermore, when combined with synephrine, which activates the β2-adrenergic receptor (β2-AR) causing relaxation of bronchial smooth muscle, stachydrine enhances the bronchodilatory effect of synephrine, thus improving its efficacy in reducing airway spasms and supporting its use in asthma treatment (Shi et al., 2009).

### 2.6 Potential activity of stachydrine analogues

Stachydrine not only has numerous pharmacological effects but also holds promise through its derivatives, which may offer superior biological activities. Modifying stachydrine by adding different functional groups can amplify its medicinal properties, increase its lipophilicity, and significantly improve its bioavailability (Zeng et al., 2023). These advancements heighten the anticipation for future applications of stachydrine and its analogues.

## 3 Functions of stachydrine in plants and bacteria

### 3.1 Functions of stachydrine in plants

Stachydrine, a prevalent secondary metabolite in plants, plays a significant role in various physiological processes. It regulates osmotic pressure, aiding plants like alfalfa in swiftly accumulating stachydrine to counteract external salt stress. This accumulation enhances the resilience of plant against salt stress (Trinchant et al., 2004). Experiments with *Arabidopsis thaliana* have shown that adding appropriate amounts of stachydrine to growth media under salt stress significantly improves plant growth.

Stachydrine activates the NodD2 protein, a key regulatory element in rhizobium-plant symbiosis. This activation facilitates the expression of nodulation genes essential for rhizome formation and nitrogen fixation, bolstering the plant’s ability to absorb and utilize nitrogen efficiently (Phillips et al., 1992; Phillips et al., 1998).

Stachydrine also enhances plant resistance to specific pests. It has been shown to impede the growth and survival of larvae from pests such as the dance moth, providing a protective benefit against these invaders (Jiang et al., 2021).

Furthermore, stachydrine plays a role in the plant’s response to abiotic stresses, such as drought and salt stress. Its involvement helps plants adapt to adverse environmental conditions, thereby enhancing their overall resilience (Phillips et al., 1992; Randall et al., 1996; Hamilton and Heckathorn, 2001; Zhang et al., 2018; Jiang et al., 2021).

Stachydrine may influence the synthesis of important secondary metabolites, such as flavonoids, which are crucial for plant defense. It also acts as a regulator of plant growth, promoting development by modulating both primary and secondary metabolic pathways (Hamilton and Heckathorn, 2001; Wu et al., 2024).

Overall, stachydrine is instrumental in regulating various physiological and ecological processes within plants, contributing significantly to their growth, development, defense mechanisms, and stress responses.

### 3.2 Role of stachydrine in bacteria

Stachydrine exerts an osmoprotective effect not only in plants and animals but also in bacteria. Research has shown that stachydrine can significantly improve the growth of *Escherichia coli* under salt stress conditions when added to its culture medium, illustrating its protective role against osmotic stress (Hanson et al., 1994). Bacillus subtilis utilizes a dedicated transporter protein for stachydrine uptake, employing it as an osmoprotectant (Horn et al., 2006). Growth studies of Bacillus subtilis further underscore the osmoprotective effects of stachydrine, which also extend to strains of *Staphylococcus aureus*, *Staphylococcus epidermidis*, and *Staphylococcus saprophyticus (* Amin et al., 1995). Notably, in the human gut—which is characterized by high osmotic pressure—stachydrine helps maintain a thriving gut flora due to its osmoprotective action on these bacteria (Chambers and Kunin, 1987a).

Stachydrine functions as an effective osmoprotective compound in bacteria, supported by specific transport systems such as BetS, Prb, and others, which actively mediate the uptake or release of stachydrine. These systems often involve ATP-binding cassette (ABC) transporter proteins or Na^+^/stachydrine cotransporter proteins that respond to changes in osmolality to regulate intracellular osmoregulator levels. Acting as a compatible solute, stachydrine accumulates in the cytoplasm at high concentrations without disrupting cellular processes, aiding in maintaining cellular water balance and alleviating osmotic pressure induced by stress (Boscari et al., 2006; Horn et al., 2006).

Furthermore, the unique molecular structure of stachydrine allows it to interact with proteins and cellular membranes, potentially stabilizing protein structures through hydrophobic or electrostatic interactions. It may also act as a molecular chaperone, assisting in proper protein folding and preventing denaturation and aggregation under conditions of high salt or drought, thus preserving intracellular protein functionality during stress. Changes in osmotic pressure can influence the expression of genes involved in osmoregulator production and transportation in bacteria. The presence of stachydrine can modulate the activity of specific genes, either stimulating or suppressing their expression, thereby regulating osmoregulator synthesis and intracellular accumulation (Chambers and Kunin, 1987b; Hanson et al., 1994; Amin et al., 1995; Alloing et al., 2006; Bashir et al., 2014).

Through these mechanisms, stachydrine acts as a molecular shield for bacteria against osmotic stress, safeguarding the structural and functional integrity of cellular components.

**Figure 3:**
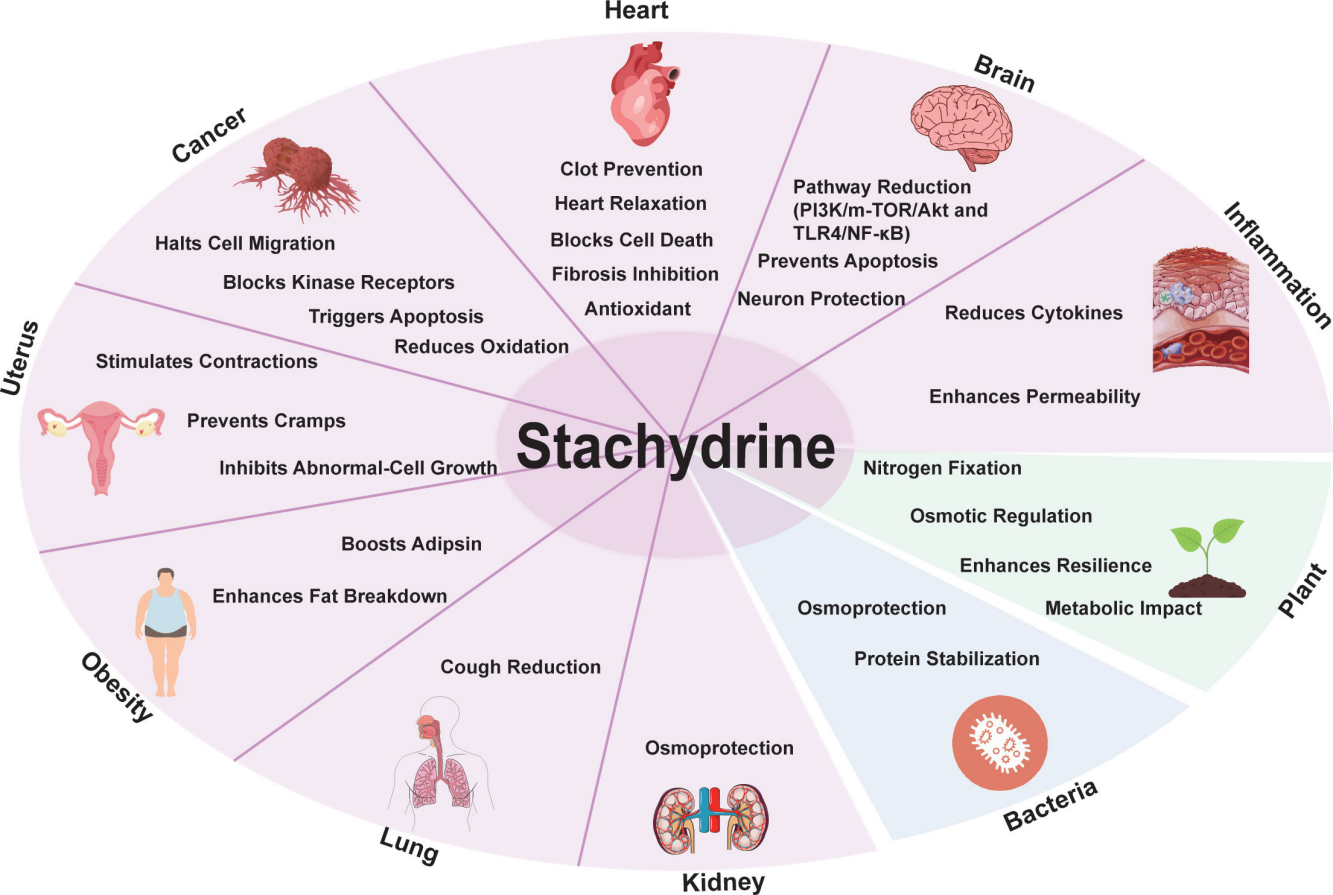
General overview of the biological activities of stachydrine. This figure illustrates the functions of stachydrine in the heart, brain, uterus, lungs, and kidneys, as well as its roles in cancer resistance and obesity; it also details the functions of stachydrine in plants and bacteria.

**Figure 3** illustrates the functions of stachydrine in humans, plants, and bacteria as described above.

## 4 Progress in the anabolism of stachydrine

### 4.1 Possible synthetic pathways for stachydrine

Research into the production process of stachydrine has revealed insights, yet the precise biological mechanism of its synthesis remains elusive. Initially, studies suggested that ornithine in alfalfa could serve as a precursor to stachydrine. Experiments with isotope-labeled ornithine demonstrated its conversion into glutamic acid and then into proline, leading to stachydrine synthesis (Morgan and Marion, 1956). Additionally, methylation, particularly from the methyl group sourced from methionine, plays a crucial role in incorporating into stachydrine’s molecular structure. It has been observed that mature alfalfa plants at 12 weeks old produce stachydrine, unlike those aged 2–3 week (Leete et al., 1955; Wiehler and Marion, 1958; Robertson and Marion, 1960; Essery et al., 1962).

**Figure 4:**
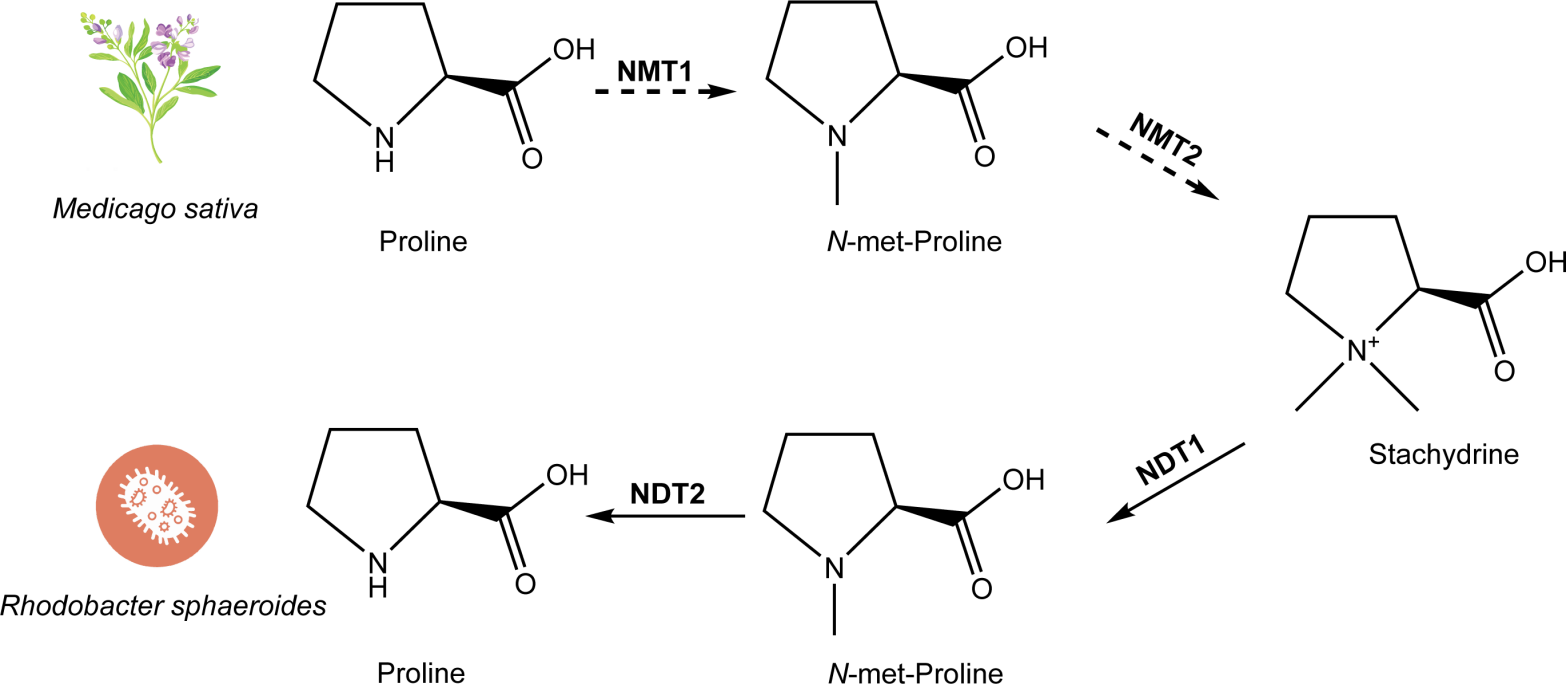
The biosynthetic and metabolic pathway of stachydrine. The biosynthetic pathway of stachydrine has been studied in *Medicago sativa*, where proline is catalyzed by NMT1 and NMT2 (which may be the same enzyme or different enzymes) through 1–2 step reactions to produce stachydrine. The degradation process of stachydrine has been studied in *Rhodobacter sphaeroides*, where stachydrine is hydrolyzed back to proline through a two-step reaction catalyzed by NDT1 and NDT2, respectively.

Chemically known as (2S)-1,1-dimethylpyrrolidine-2-carboxylic acid or N, N-dimethyl-L-proline, stachydrine is believed to be synthesized through a process involving the addition of two methyl groups to the N of proline (**Figure 4**). Various substrates, including iodomethane and dimethyl sulphate, have been used to react with L-proline to synthesize stachydrine, aiming to introduce two methyl groups to the N of proline (He and Wang, 2005a; He and Wang, 2005b). Experiments with proline containing a radioactive isotope in alfalfa plants led to the detection of radioactive N-methyl proline and stachydrine, suggesting the catalytic activity of N-methyltransferase (NMT) in this synthesis.

Despite advancements, studies into the biosynthesis of stachydrine are limited. Currently, stachydrine is produced either by extraction from plants or by chemical synthesis. Unraveling its biosynthetic pathway could significantly advance phytopharmacology, aiding in understanding stachydrine’s mechanism of action and interactions, potentially enhancing drug quality control, and facilitating the development of new pharmaceuticals through synthetic biology and genetic engineering.

### 4.2 Metabolic pathway of stachydrine

The metabolic pathways of stachydrine are largely understood, although its biosynthesis pathway remains unelucidated (**Figure 4**). Stachydrine is excreted by germinating seeds and plant roots into bacterial environments. Bacteria closely associated with plants have evolved mechanisms to utilize betaine for two primary purposes: as a protective shield against high osmotic pressure and as a source of carbon and nitrogen in the absence of osmotic stress. Recent research has identified the metabolic pathway of betaine in two strains of bacteria: *Paracoccus denitrificans* and *Rhodobacter sphaeroides*. Initially, stachydrine is enzymatically converted into *N*-methyl stachydrine by the action of the first *N*-desmethyltransferase enzyme (NDT1). Subsequently, proline is synthesized through the activity of the second *N*-desmethyltransferase enzyme (NDT2). Finally, proline undergoes further metabolism into glutamic acid (Kumar et al., 2014) (**Figure 4**). Notably, the metabolic pathway of stachydrine is essentially the reverse of the proposed synthetic pathway of stachydrine.

## 5 Outlook

Stachydrine, a versatile bioactive molecule, has garnered significant interest due to its promising medicinal properties. Ongoing research is dedicated to elucidating its precise mechanisms of action, refining its applications, and exploring its potential in pharmaceutical development. A key focus of these efforts is understanding the biosynthesis pathway of stachydrine. Despite the apparent simplicity of converting proline to stachydrine, research on this synthesis pathway in alfalfa has been challenging since the 1950s, primarily due to the lack of relevant enzyme identification. This challenge may be attributed to the limited availability of genomic data and the nascent stage of molecular biology techniques at that time. Alternatively, the complexity of the reaction in organisms may require more advanced genomic and multifaceted biochemical experiments for confirmation.

The initial exploration of the biosynthesis route of stachydrine is now being modeled using bioinformatics tools and systems biology approaches. This theoretical analysis helps predict unidentified intermediates and key enzymes, providing a foundation for experimental design. Radioisotope or stable isotope labeling techniques are also employed to trace the biosynthetic pathway of stachydrine, offering a detailed understanding of each step in the synthesis process.

Understanding the biochemical pathway of stachydrine paves the way for using genetic engineering to manipulate or enhance the genes involved in its biosynthesis in plants, potentially increasing stachydrine production. Synthetic biology opens possibilities for creating microbial cell factories that express plant-derived enzymes, enabling the production of stachydrine or its precursors. Metabolic engineering strategies aim to optimize metabolic pathways in plants or microorganisms to improve stachydrine biosynthesis, reduce by-product formation, and enhance the yield and purity of desired molecules.

Furthermore, the exploration of stachydrine and its derivatives through high-throughput screening and natural product mining may uncover novel analogues from other plants or microorganisms. These analogues might exhibit improved pharmacological activities or decreased toxicity compared to stachydrine itself. Additionally, integrating biosynthetic pathways from diverse organisms could lead to the generation of novel stachydrine derivatives using combinatorial biosynthesis techniques, thus expanding the spectrum of chemical variations and potential therapeutic applications.

In summary, a deeper understanding of the biosynthetic process of stachydrine promises to significantly advance its utilization in medicine, health, and agriculture, opening new avenues for research and development in these fields.

## Author contributions

ZH: Conceptualization, Data curation, Formal analysis, Investigation, Methodology, Project administration, Resources, Software, Validation, Visualization, Writing – original draft, Writing – review & editing. PL: Writing – review & editing, Methodology, Project administration, Resources. PL: Writing – review & editing. PX: Data curation, Formal analysis, Funding acquisition, Project administration, Resources, Supervision, Visualization, Writing – review & editing, Methodology, Writing – original draft.
